# Transcriptomic Analysis of *Fusarium verticillioides* Across Different Cultivation Periods Reveals Dynamic Gene Expression Changes

**DOI:** 10.3390/microorganisms14010102

**Published:** 2026-01-02

**Authors:** Meng-Ling Deng, Jun-Jun He, Xin-Yan Xie, Jian-Fa Yang, Fan-Fan Shu, Feng-Cai Zou, Lu-Yang Wang, Jun Ma

**Affiliations:** 1 Faculty of Animal Science and Technology, Yunnan Agricultural University, Kunming 650201, China; merlideng@126.com (M.-L.D.); zfc1207@vip.163.com (F.-C.Z.); 2 The Yunnan Key Laboratory of Veterinary Etiological Biology, College of Veterinary Medicine, Yunnan Agricultural University, Kunming 650201, China; hejunjun617@163.com (J.-J.H.); zzqq6188@outlook.com (X.-Y.X.); jsc315@163.com (J.-F.Y.); shuff1227@163.com (F.-F.S.)

**Keywords:** *Fusarium verticillioides*, dynamic transcriptomic profiling, gene expression, cultivation periods

## Abstract

*Fusarium verticillioides* is a common pathogenic fungus of corn since it causes severe yield losses and produces mycotoxins to threaten the health of both humans and livestock. Although extensive research has characterized specific genetic and environmental factors influencing mycotoxin production, a systematic understanding of the temporal transcriptional dynamics governing its developmental progression remains lacking. This study addresses this critical knowledge gap through a time-series transcriptomic analysis of *F. verticillioides* at four key cultivation stages (3, 5, 7, and 9 days post-inoculation). Transcriptomic analysis identified 1928, 2818, and 1934 differentially expressed genes (DEGs) in the comparisons of FV3 vs. FV5, FV5 vs. FV7, and FV7 vs. FV9, respectively. Gene Ontology enrichment revealed 76, 106, and 56 significantly enriched terms across these comparisons, with “integral component of membrane” consistently being the most enriched cellular component. Pathway analysis demonstrated “amino acid metabolism” and “carbohydrate metabolism” as the most significantly enriched metabolic pathways. Notably, the fumonisin (FUM) and fusaric acid (FA) biosynthetic gene clusters exhibited coordinated peak expression during the early cultivation, followed by progressive decline. Mfuzz clustering further delineated 12 distinct expression trajectories, highlighting the dynamic transcriptional networks underlying fungal adaptation. This work provided the first comprehensive temporal transcriptome of *F. verticillioides*, establishing a foundational resource for understanding its stage-specific biology and revealing potential time-sensitive targets for future intervention strategies.

## 1. Introduction

*Fusarium verticillioides* is one of the most widespread fungal pathogens of corn, causing seedling rot, ear rot, and stalk rot. Its pathogenicity leads to devastating yield losses and reduction in corn quality [1,2,3,4]. Additionally, *F. verticillioides* has been confirmed as a pathogen of humans, especially to individuals with compromised immune systems, including those with primary immunodeficiencies, undergoing chemotherapy, or having received hematopoietic cell transplantation [5]. Furthermore, *F. verticillioides* produces various types of mycotoxins, like fumonisins, fusaric acid, and miniliformin [6]. Fumonisins are the most abundant mycotoxins found in contaminated maize, among which fumonisin B1 is of primary concern due to its acute toxicity to livestock and humans [7,8], which causes several fatal diseases, such as cancers, birth neural defects, and other related organ function impairments [9,10,11,12]. Fusaric acid (FA) is another toxin produced by *F. verticillioides*, which synergistically enhances the toxicity of other *Fusarium* mycotoxins [13] and is hazardous to plants, humans, and other animals [14]. Consequently, devising effective strategies to mitigate both crop damage and mycotoxin contamination remains a major challenge for agricultural and food security. Extensive research has elucidated pathogenicity and host interactions [1,4], environmental adaptability [15], mycotoxin biosynthesis [10,13,16], molecular biology, and gene functions [17,18,19,20]. However, the prevailing research has largely relied on comparative studies under specific stress conditions or single-time-point analyses, resulting in a critical knowledge gap: the absence of a systematic, time-resolved understanding of the transcriptional dynamics governing *F. verticillioides* development under standard cultivation conditions. This gap hinders the identification of key regulatory nodes and stage-specific vulnerabilities that could be exploited for precise, timely intervention, moving beyond broad-spectrum antifungal approaches.

RNA-Seq is a high-throughput sequencing technology that enables comprehensive acquisition of dynamic gene expression patterns associated with critical developmental stages (such as spore germination, hyphal growth, and conidiation), laying a basis to elucidate the alterations of cellular growth characteristics, pathways, and functions in *F. verticillioides* during cultivation. In recent years, RNA-Seq has been a powerful technology in mycological research. Fungi, such as *Tremella aurantialba* [21], *Aspergillus oryzae* [22], *Agaricus bisporus* [23], and *Coprinopsis cinerea* [24], have been extensively studied by RNA-Seq. These efforts have significantly advanced our understanding of the functional genes involved in fungal growth and developmental mechanisms, shedding light on the complex biological processes that underpin fungal life cycles. Despite these advances, the fundamental knowledge regarding the dynamics of biological processes, transcriptomes, and metabolic pathways across different cultivation periods remains fragmentary.

In this study, we performed time-series RNA sequencing on *F. verticillioides*, sampled at four strategic time points—3, 5, 7, and 9 days post-inoculation—representing key phases from early growth to the late stationary stage. This allowed us to establish the first high-resolution temporal transcriptome over a full cultivation cycle, to delineate phase-specific alterations in functional modules and metabolic pathways, and to define the expression kinetics of the fumonisin (FUM) and fusaric acid (FA) biosynthetic gene clusters in relation to overarching developmental regulation.

## 2. Materials and Methods

### 2.1. Sample Preparation Growth Curve Measure

The *F. verticillioides* RMYN1 strain utilized in this study was isolated from the naturally infected *Rhipicephalus microplus* tick in Yunnan Province, China. It is preserved in the culture collection of the Yunnan Key Laboratory of Veterinary Etiological Biology (GenBank accession number: PQ012594). The strain was cultured on potato dextrose agar (PDA) for 72 h, then 5 mm mycelium was collected and transferred to the center of fresh PDA medium and incubated at 28 °C in complete darkness. To characterize the growth kinetics, the colony diameter was measured daily for six days. Concurrently, for biomass determination, fungal mycelia were harvested, dried overnight, and weighed daily over a ten-day period. Based on the biomass and growth curves, samples at 3 days, 5 days, 7 days, and 9 days post-cultivation were collected for transcriptome analysis, and three replicates were set for each time point.

### 2.2. Total RNA Extraction and Transcriptomic Sequencing

The total RNA of each sample was extracted by using the Trizol method. RNA concentration and purity were determined by NanoDrop 2000 (Thermo Fisher Scientific, Wilmington, DE, USA) to ensure that the absorbance ratio of 260 nm/280 nm between 1.8 and 2.2, and the absorbance ratio of 260 nm/230 nm greater than or equal to 2.0. RNA integrity was assessed with an RNA Nano 6000 Assay Kit on the Agilent Bioanalyzer 2100 system (Agilent Technologies, Santa Clara, CA, USA). RNA-seq library construction and RNA sequencing were performed by Biomarker Technologies Co., Ltd. (Beijing, China).

### 2.3. RNA-Seq Analysis and Gene Function Annotation

To ensure data quality and reliability for subsequent analysis, FastQC software (v0.12.1) was used for the filtering of raw reads, and the clean data were obtained by removing sequencing adapters, the reads containing poly-N, and the low-quality reads from raw data. The transcriptomic analysis was conducted by using the *F. verticillioides* 7600 reference genome and annotation files (accession number: GCF_000149555) that were downloaded from NCBI databases. Clean reads were mapped to reference the *F. verticillioides* genome, using Hisat2 software (v2.0.5) [25]. Transcript assembly, alternative splicing prediction, and gene model refinement were performed using StringTie (v2.2.1) [26], based on the alignment results. DIAMOND (v 2.0.15) [27] was used to annotate the genes via the following databases: COG (Clusters of Orthologous Groups), GO (Gene Ontology), KEGG (Kyoto Encyclopedia of Genes and Genomes), KOG (euKaryotic Orthologous Groups), Pfam (Protein family), Swiss-Prot (a manually annotated and reviewed protein sequence database), eggNOG (Evolutionary Genealogy of Genes: Non-supervised Orthologous Groups), and NR (NCBI nonredundant database).

In this study, gene expression levels were measured using the FPKM (fragments per kilobase of transcript per million mapped reads) method. Differential expression analysis was conducted by using the DESeq2 R package (v1.30.1). The differentially expressed genes (DEGs) were identified with the threshold ${|\log}_{2}\left(Fold\;change\right)|\geq 1$ and FDR (false discovery rate) < 0.01. Functional enrichment analysis of DEGs was performed by using ClusterProfiler packages (v4.4.4)and KOBAS-i (v3.0) [28]; the significance level was determined by *p* < 0.05. The expression differences in the fumonisin (FUM) and fusaric acid (FA) biosynthetic gene clusters across various cultivation periods were analyzed, and heatmaps were generated with the R package ComplexHeatmap (v2.18.0).

### 2.4. Time Series Gene Clustering

To examine the gene time-series expression pattern of *F. verticillioides*, the DEGs from all comparison groups were normalized and clustered by the Mfuzz R package (v2.66.0) which conducts soft clustering based on the fuzzy c-means algorithm. The number of clusters was set to 12. The fuzzifier coefficient of variation and membership values was set to >0.5 [29]. Subsequently, DEGs within each cluster were subjected to GO and KEGG pathway enrichment analysis using the ClusterProfiler packages and KOBAS-i, with a significance threshold of *p* < 0.05.

### 2.5. Real-Time Quantitative PCR Validation of DEGs

To verify the DEGs identified from transcriptomic analysis, RT-qPCR was performed, and *actin* served as the reference gene to normalize the gene expression. RT-qPCR was carried out with the PerfectStart^®^ Green qPCR SuperMix (TransGen Biotech, Beijing, China) and Bio-Rad CFX96. Primers used in the RT-qPCR experiment are shown in Supplementary Table S1. Negative control reactions were conducted to avoid contamination, and gene expression levels were normalized to *actin*. Each sample was amplified in triplicate. The $2^{\left(-\Delta \Delta Ct\right)}$ method was employed to calculate the relative expression level [30].

## 3. Results

### 3.1. Growth Dynamics of F. verticillioides

The in vitro growth of *F. verticillioides* was assessed by colony expansion and biomass accumulation. The results showed that the mycelia grew fastest at 4 days (Supplementary Figure S1A), which was consistent with a previous study [31]. The biomass accumulation showed a rapid increase from day 3 to day 5, with the highest growth rate occurring on day 5. Growth slowed thereafter, and the maximum biomass was reached by day 9, marking entry into the stationary phase (Supplementary Figure S1B).

### 3.2. Overview of RNA-Seq Data

To systematically identify the spatiotemporal gene expression profiles of *F. verticillioides* during cultivation, RNA sequencing was used to analyze mycelia collected at four time points—3, 5, 7, and 9 days—which corresponded to the early exponential phase, peak biosynthetic activity, transitional phase, and early stationary phase, respectively, based on the growth dynamics (Supplementary Figure S1). Approximately 79.38 Gb of high-quality clean bases were obtained, with the GC content ranging from 51.33 to 52.19% and all Q30 values exceeding 93.79% (Table 1). Over 91% of the clean reads from each sample were aligned to the reference genome of *F. verticillioides* (Table 1). These statistical results indicated the high RNA-seq quality for subsequent analysis. In total, 17,365 genes were identified.

**Figure 1:**
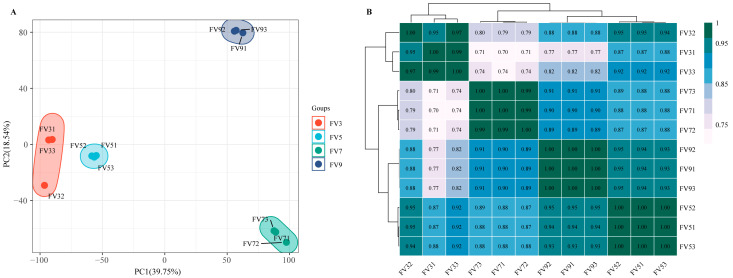
The global transcriptomic character of *Fusarium verticillioides* across different cultivation periods. (**A**) The results of PCA. (**B**) The results of correlation analysis. Samples at 3, 5, 7, and 9 days post-cultivation were designated as FV3, FV5, FV7, and FV9 (three replicates each; e.g., FV31–FV33 for day 3).

Principal component analysis (PCA) revealed clear separation of the samples into four distinct clusters corresponding to the four time points (Figure 1A). Notably, the day 3 and day 5 samples clustered in close proximity to each other. Analysis of global gene expression correlations demonstrated strong intra-group transcriptomic similarity (R^2^ > 0.95), whereas inter-group comparisons showed substantially lower correlation coefficients (R^2^ ≈ 0.70) (Figure 1B). Together, these findings indicate distinct gene expression profiles between the early (3–5 days) and late (7–9 days) growth stages of *F. verticillioides*.

### 3.3. Functional Annotation and Classification of the Unigenes

A total of 16,098 unigenes were annotated against eight public databases, including 5539 (31.9%) in the KOG database, 5603 (32.3%) in the COG database, 7042 (40.6%) in the Swiss-Prot database, 8122 (46.8%) in the KO database, 9788 (56.4%) in the GO database, 10,518 (60.6%) in the Pfam database, 10,635 (61.2%) in the eggNOG database, and 16,096 (92.7%) in the NR database. Intersectional analysis showed that 2938 genes were annotated by all eight databases.

**Figure 2:**
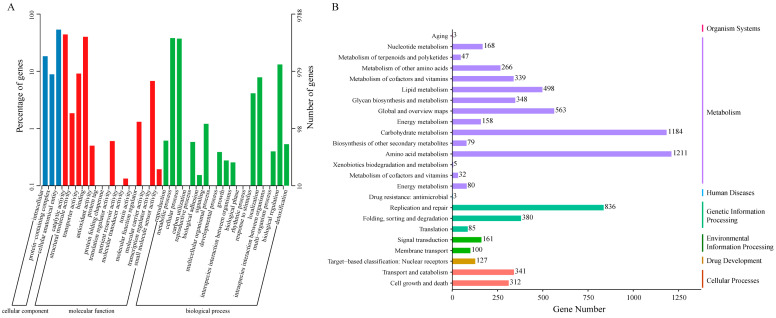
Gene functional annotation, based on the GO (**A**) and KEGG (**B**) pathway databases.

GO analysis classified the 9788 annotated genes into three primary GO ontologies, including biological process (BP), molecular function (MF), and cellular component (CC) (Figure 2A). In the BP group, the dominant terms enriched with the most genes were “metabolic process”, “cellular process”, and “biological regulation”, which contained 3725, 3641, and 1289 genes, respectively. In the MF group, “catalytic activity” consisted of the largest number of genes (4314), followed by “binding” (3926 genes), and “transporter activity” (662 genes). In the CC group, 5226 genes were annotated in the “cellular anatomical entity”, 1798 genes were annotated in “intracellular”, and 867 genes were related to the “protein-containing complex”.

Among the identified genes, 8122 were successfully annotated by the KEGG database. As shown in Figure 2B, a large number of these genes were involved in metabolism-related pathways, including “amino acid metabolism”, “carbohydrate metabolism”, “global and overview maps”, and “lipid metabolism”. Notably, the functional categories of “amino acid metabolism”, “carbohydrate metabolism”, “replication and repair”, “global and overview maps”, and “lipid metabolism” emerged as the top five most enriched pathways, highlighting the pivotal role of these gene expressions in governing the life of *F. verticillioides*.

### 3.4. Identification of DEGs Across Various Cultivation Periods

**Figure 3:**
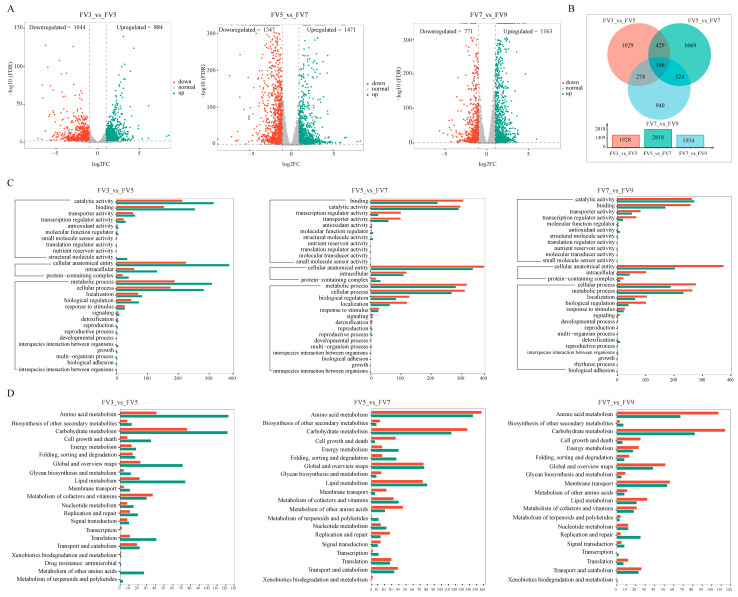
The differential expression genes analysis. (**A**) Volcano plot of DEGs. Upregulated genes, downregulated genes, and genes without significant change are shown as green, red, and gray dots, respectively. (**B**) Venn diagram of DEGs. (**C**) The GO enrichment analysis of DEGs. (**D**) The KEGG enrichment of DEGs in FV3 vs. FV5, FV5 vs. FV7, and FV7 vs. FV9, respectively. The color bar denotes the number of enriched genes, with red and green representing up- and down-regulated genes, respectively.

A total of 5061 DEGs were obtained by comparison with adjacent stages (Supplementary Table S2). The FV3 vs. FV5 comparison yielded 1928 DEGs (884 upregulated and 1044 downregulated). For the group FV5 vs. FV7, 2818 DEGs were found, including 1471 upregulated genes and 1347 downregulated. In the FV7 vs. FV9 group, 1934 DEGs were identified (1163 upregulated and 771 downregulated) (Figure 3A). The proportion of downregulated genes decreased continuously from 54.1% (FV3 vs. FV5) to 47.8% (FV5 vs. FV7) and 39.9% (FV7 vs. FV9), indicating a reduction in overall metabolic activity, consistent with the culture entering a later, more stable phase. Venn diagram analysis showed that only 196 DEGs were shared among three comparison groups, with the FV5 vs. FV7 exhibiting the largest number group-specific genes (1669 genes) (Figure 3B), confirming that the transcriptional response was highly stage-specific. Collectively, these results demonstrated that distinct cultivation periods exhibit a unique transcriptomic profile.

### 3.5. Functional Annotation of DEGs

To characterize the biological differences in *F. verticillioides* among different cultivation periods, the functional enrichment analysis of DEGs was performed. In FV3 vs. FV5, DEGs were significantly enriched in 76 GO terms, comprising 43 BP, 8 CC, and 25 MF terms. The “integral component of membrane” (GO:0016021), “translation” (GO:0006412), “structural constituent of ribosome” (GO:0003735), “citrate metabolic process” (GO:0006101), and “high-affinity iron permease complex” (GO:0033573) were the top five most significantly enriched terms (Figure 3C). For FV5 vs. FV7, DEGs were assigned into the total 106 GO terms (71 BP, 4 CC, 31 MF), the top five enriched terms were “transcription, DNA-templated” (GO:0006351), “zinc ion binding” (GO:0008270), and “DNA-binding transcription factor activity, RNA polymerase II-specific” (GO:0000981), “integral component of membrane” (GO:0016021), and “host cell nucleus” (GO:0042025) (Figure 3C). Finally, in the FV7 vs. FV9 group, DEGs were significantly categorized into 56 GO terms (25 BP, 6 CC, 25 MF). The top five enriched terms here were “integral component of membrane” (GO:0016021), “carbohydrate transport” (GO:0008643), “oxidoreductase activity” (GO:0016491), “regulation of cyclin-dependent protein serine/threonine kinase activity” (GO:0000079), and “transmembrane transporter activity” (GO:0022857). Additionally, the integral component of the membrane was the most enriched term in all three comparisons. The details about GO enrichment are listed in Supplementary Table S3.

In addition, we also performed KEGG analysis to further investigate the biological roles of these DEGs. As shown in Figure 3D, “amino acid metabolism” and “carbohydrate metabolism” were significantly enriched in all three comparison groups. Notably, in the FV3 vs. FV5 group, these two pathways contained more downregulated than upregulated DEGs, but the opposite pattern was seen in FV5 vs. FV7 and FV7 vs. FV9 (Figure 3D). Among the most significantly enriched pathways, “Ribosome”, “Biosynthesis of amino acids” and “Glycine, serine, and threonine metabolism” contained the greatest number of DEGs at the early shift (FV3 vs. FV5) (Supplementary Figure S2). Between 5 days and 7 days, the top three pathways with the largest DEGs numbers were “Carbon metabolism”, “Glycolysis/Gluconeogenesis” and “Glycine, serine and threonine metabolism” (Supplementary Figure S2). Interestingly, “Glycolysis/Gluconeogenesis” and “Carbon metabolism” remained among the top enriched pathways during the final shift (FV7 vs. FV9) (Supplementary Figure S2). These results suggested that distinct cultivation periods display divergent metabolic pathways. While each stage exhibited a unique transcriptional profile, the persistent enrichment of the amino acid and carbohydrate metabolism underscores their fundamental role throughout the cultivation cycle of *F. verticillioides*.

### 3.6. Expression Pattern of FUM Cluster Genes and FA Cluster Genes

**Figure 4:**
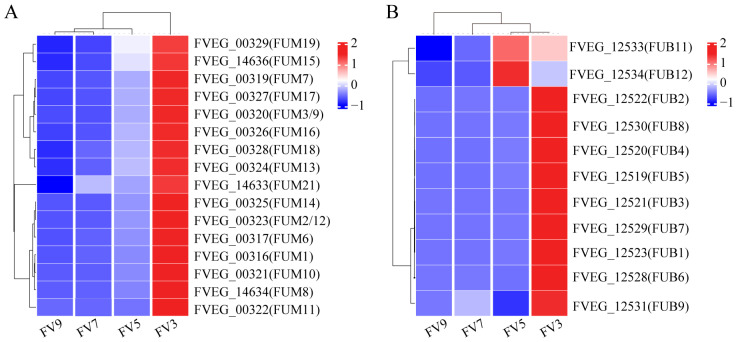
Heat map of expression of fumonisin gene cluster (**A**) and fusaric acid gene cluster (**B**). Dotted lines indicate sample-group and gene-cluster boundaries.

To elucidate the synthesis patterns of fumonisins and fusaric acid, we also focused on the expression of genes involved in their biosynthetic gene clusters. The FUM cluster included 17 genes [15], of which 16 were observed to be differentially expressed, and all showed higher levels at the early time point (day 3) followed by a gradual decline throughout cultivation (Figure 4A). The FA biosynthetic cluster included 12 genes [16,17]: with the exception of FVEG_12532 (*FUB10*), the remaining 11 genes were differentially expressed and exhibited a temporal pattern similar to that of the FUM cluster (Figure 4B). Notably, the transcriptional profiles of *FUB11* and *FUB12* were distinct from the other nine cluster genes, suggesting that they may function independently of the core biosynthetic pathway. The synchronous decline in expression of both toxin clusters is consistent with a global reduction in metabolic activity, indicating a diminished transcriptional commitment to fumonisin and fusaric acid biosynthesis during the shift to a stable phase.

Other than the FUM cluster, we analyzed the expression of genes involved in fumonisin production regulation (Table 2). Differential expression analysis revealed that these genes showed distinct transcriptional patterns across different cultivation periods. In the early shift (FV3 vs. FV5), 21 genes were downregulated and only 5 were upregulated. Conversely, there were more upregulated genes and fewer downregulated genes in FV5 vs. FV7 and FV7 vs. FV9. The genes encoding a putative zinc-binding dehydrogenase (*FvZBD1*) were significantly downregulated during the early period (3–5 days) but upregulated in the later growth stages (7–9 days). Furthermore, the MFS transporter-encoding gene *FST1* exhibited consistent downregulation across all comparisons (Table 2), strongly supporting its global negative regulator role in fumonisin biosynthesis, potentially through the modulation of precursor transport or signaling pathways.

### 3.7. Clustering Analysis of Time Series Genes

**Figure 5:**
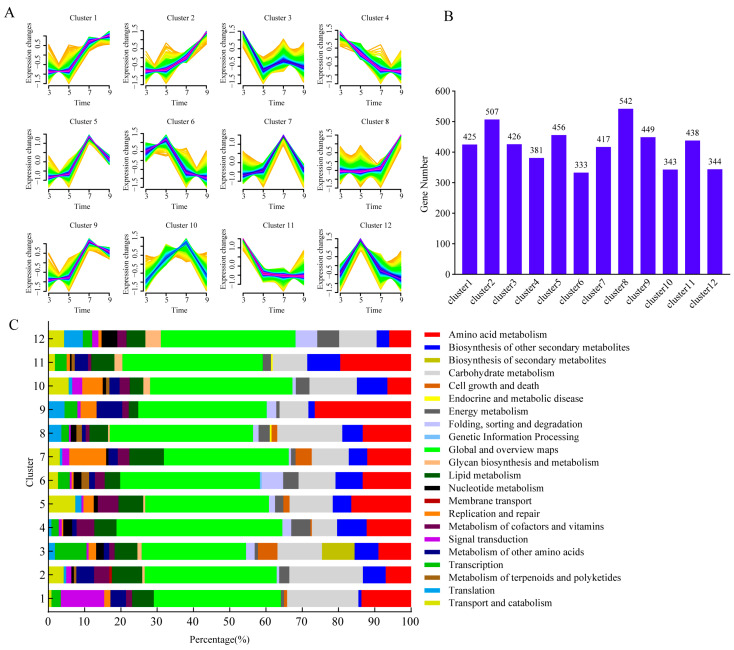
Cluster of gene expression profiles across different growth stages. (**A**) Performing Mfuzz clustering analysis on DEGs; The yellow and green lines corresponded to genes with low membership values, while the red and blue lines corresponded to genes with high membership values. (**B**) The number of genes of each cluster. (**C**) Functional annotation of each gene cluster.

To delineate expression dynamics across cultivation, we performed time-series clustering on the 5061 DEGs. All DEGs were grouped into 12 time-specific clusters, with the number of genes in each group ranging from 333 to 542 (Figure 5A,B). Cluster 2 and 4 exhibited an opposite expression pattern, the cluster 2 showed a gradual increase from 3 days to 9 days post-cultivation, while cluster 4 genes decreased overtime, which suggested these genes in cluster 2 mainly involved in early cultivation stage and these transcripts in cluster 4 were potentially involved in a later cultivation stage. Functional annotation revealed that cluster 2 was primarily enriched in “Global and overview maps” and “Carbohydrate metabolism”, while cluster 4 was mainly related to “Global and overview maps” and “Amino acid metabolism” (Figure 5C). Similarly, cluster 8 displayed a steady increase until day 7, followed by a sharp rise at day 9, contrasting with the declining trend of cluster 11, indicating divergent regulatory roles in *F. verticillioides* development. Cluster 5 and 9 exhibited a similar tendency with increased gradually and sharply from day 3 to day 5 and day 7, respectively, and the expression level reached its highest level on day 7, then subsequently decreased on day 9. These findings highlighted dynamic gene expression networks underlying *F. verticillioides* growth and metabolic adaptation.

### 3.8. RT-qPCR Validation

**Figure 6:**
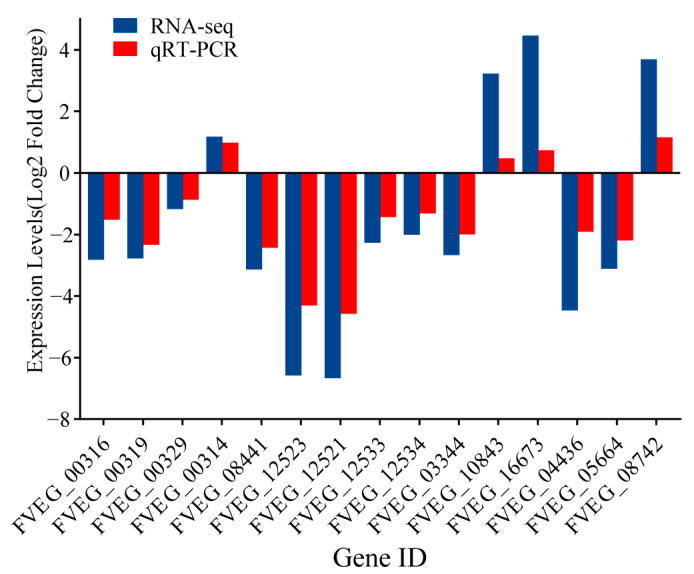
Validation of DEGs by RT-qPCR.

The reliability of the RNA-seq data was validated using RT-qPCR method. Representative genes FVEG_00316 (FUM1), FVEG_00319 (FUM7), FVEG_00329 (FUM19), FVEG 00314 (FvZBD1) and FVEG 08441 (FST1) involved in fumonisins synthesis and regulation, FVEG_12523 (FUB1), FVEG_12521 (FUB3), FVEG_12533 (FUB11), FVEG_12534 (FUB12) related to FA synthesis, and other FVEG_01440, FVEG_10843, FVEG_04436, FVEG_16673, FVEG_05664, and FVEG_08742 were selected for validation. The RT-qPCR results corroborated the RNA-seq findings, further confirming the reliability of the expression profiles (Figure 6).

## 4. Discussion

Fungal developmental genes are known to regulate diverse processes, including stress response [8,31], host–pathogen interactions [32,33], and secondary metabolism [19,34], as previously demonstrated under various experimental or stress conditions. However, the dynamic gene expression landscape of *F. verticillioides* across different cultivation time points remains unexplored. Here, we present the first transcriptome analysis of *F. verticillioides* during unperturbed, in vitro cultivation, revealing dynamic gene expression patterns in a near-physiological context.

To characterize the biological differences across different cultivation periods of *F. verticillioides*, GO and KEGG pathway enrichment analyses of DEGs were conducted. As shown in Figure 3, these DEGs were mainly enriched in membrane components and transport, energy metabolism, and material and structural metabolism. The significant enrichment of the term “integral component of membrane” (GO:0016021) in three comparison groups, “transmembrane transporter activity” (GO:0022857) enriched in FV5 vs. FV7 and FV7 vs. FV9 (Figure 3C), suggested dynamic remodeling of the plasma membrane during fungal development. This was further supported by the specific enrichment of the “fatty acid degradation” pathway and “fatty acid biosynthesis” in KEGG pathway (Supplementary Figure S2), indicating active regulation of lipid composition, a key factor in maintaining membrane fluidity and adaptability [35]. Given that the cell membrane is a primary target for antifungal strategies [36,37], our findings highlighted the potential for designing stage-specific control measures by targeting these dynamic membrane components. In phytopathogenic fungi, such as *F. verticillioides*, ABC transporters contain both uptake and efflux transport systems for fundamental cellular processes, pathogenesis, and tolerance against various toxic or xenobiotic compounds [38]. Additionally, the ABC transporter was involved in the extracellular export of fumonisins [39]. The significant enrichment of the ABC transporters pathway in FV5 vs. FV7 and FV7 vs. FV9 suggested that it could possess particular relevance for the growth of *F. verticillioides*, and this significant enrichment would therefore be related to the accumulation of fumonisins.

In KEGG analysis, the DEGs in the FV3 vs. FV5 comparison were most significantly enriched in the “Ribosome”, “Biosynthesis of amino acids”, and “Glycine, serine, and threonine metabolism” pathways (Supplementary Figure S2). This pattern strongly indicates a physiological state prioritizing rapid biomass accumulation during the early cultivation period. The marked enrichment of “Ribosome” [40], coupled with active amino acid biosynthesis, reflected an increased demand for translational machinery and protein precursors to support vigorous hyphal expansion and structural establishment, fitting the primordial growth demands of the fungus. A distinct metabolic shift was observed in the subsequent stages. In comparisons FV5 vs. FV7 and FV7 vs. FV9, DEGs were primarily enriched in the “Carbon metabolism” and “Glycolysis/Gluconeogenesis” pathways (Supplementary Figure S2). This transition from protein synthesis to central carbon metabolism suggested a reprogramming of cellular priorities from primary growth to energy production and metabolic diversification. The energy generated here may not only sustain continued growth but also, crucially, fuel the biosynthesis of secondary metabolites, including potential virulence factors. The higher degree of overlap in enriched pathways between FV5 vs. FV7 and FV7 vs. FV9, compared to FV3 vs. FV5, further highlighted this coherent biological shift, marking a distinct developmental phase. Collectively, these sequential enrichment profiles depicted a lifecycle strategy in *F. verticillioides*: an initial phase dedicated to colonial establishment (3 days to 5 days), followed by a phase geared towards metabolic preparation and potential resource allocation for virulence (5 days to 9 days).

*F. verticillioides* is recognized as a prolific producer of over fifty fumonisins. These mycotoxins are well known for their carcinogenic potential, and have drawn significant scientific attention due to their frequent contamination of cereals and associated processed food products [10]. Previous studies demonstrated that the production of fumonisins was highly dependent on the transcriptional activity of the *FUM* gene cluster [41], and environmental factors or genetic variations could influence the final toxin levels by regulating the expression of these genes [42]. Our time-resolved transcriptome data revealed a clear temporal pattern in the expression of this biosynthetic machinery. The e *FUM* cluster varied at different cultivation periods, including the pivotal gene *FUM1* (encoding the polyketide synthase and catalyzing the first step in the biosynthesis of fumonisins) [41,43] and exhibited peak transcript abundance at the earliest time point (3 days post-inoculation), followed by a gradual decline over time (Figure 4A). This expression pattern correlated with the activity of known regulatory pathways [44]. For instance, the zinc-binding dehydrogenase gene (*FvZBD1*), which has been identified as a negative regulator of fumonisin biosynthesis [18], was significantly downregulated in FV3 vs. FV5 but upregulated in FV7 vs. FV9, aligning with the elevated expression of *FUM* genes during early cultivation phases. These data suggested that the biosynthesis of fumonisin was active at the early cultivation stage, and as the cultivation time increased, the activity of fumonisin biosynthesis gradually decreased. In summary, our findings demonstrated a distinct temporal regulation pattern of fumonisin biosynthetic genes in *F. verticillioides*, with peak transcriptional activity occurring early in the cultivation cycle, and the expression pattern analysis of genes involved in fumonisin regulation revealed a complex biosynthetic network.

FA is another key mycotoxin produced by *F. verticillioides*, which is notable for its high phytotoxicity [45,46,47] and moderate toxicity to humans and animals [14,48,49,50,51]. Our study showed that the majority of genes in the gene cluster of FA biosynthetic exhibited a similar pattern to the FUM cluster (Figure 4B); this coordinated transcriptional dynamics strongly suggests a potential functional interaction or co-regulation between these two metabolic pathways, which may underlie their documented synergistic toxicity and enable *F. verticillioides* to initiate co-synthesis of both mycotoxins. Although FA biosynthesis directly regulates the FUM cluster or vice versa remains an open question; the coordinated expression of these genes of *F. verticillioides* likely aids us to establish a multi-faceted foundation for host infection, colonization, and environmental adaptation. Future studies employing genetic approaches, such as targeted deletion of key regulators in one cluster while monitoring the outcome in the other, will be essential to disentangle causality and elucidate the precise nature of this coordinated expression. Moreover, we found that the transcription timing of *FUB11* and *FUB12* was different from the others, which may reflect different roles of these two sets of genes. We inferred that *FUB11* and *FUB12* were involved in the export of FA, while the other cluster genes were responsible for endogenous production of FA [17]. A previous study reported that ∆fub12 mutants accumulated FA to only 25% of the wild-type level [17], which may support our hypothesis.

## 5. Conclusions

This study provides the first time-series transcriptomic atlas of *F. verticillioides* across four key in vitro cultivation time points (3, 5, 7, and 9 days), delineating the dynamic gene expression patterns underlying its developmental progression. We identified the amino acid and carbohydrate metabolisms as central metabolic pathways throughout the cultivation stages and revealed a coordinated peak expression of fumonisin and fusaric acid biosynthetic gene clusters. These findings systematically illuminate the temporal regulatory logic of fungal growth and mycotoxin production. The dataset established here serves as a foundational resource for understanding the stage-specific pathogenicity of *F. verticillioides* and highlights key metabolic pathways and toxin synthesis time windows as potential targets for timely and precise intervention strategies against *F. verticillioides*.

## Figures and Tables

**Table 1:** Transcriptomic sequencing quality and mapping data of each *F. verticillioides* sample.

Sample ID	Clean Reads	Clean Bases (Gb)	Q20	Q30	GC Content	Total Mapped	Uniquely Mapped
FV31	43324608	6.48	97.98%	94.50%	52.05%	92.53%	92.21%
FV32	50160432	7.51	97.85%	94.28%	52.19%	91.82%	91.42%
FV33	40044176	6.00	97.92%	94.34%	52.13%	92.93%	92.64%
FV51	44039586	6.59	97.97%	94.46%	51.84%	94.07%	93.65%
FV52	45426032	6.79	98.19%	95.03%	51.65%	94.09%	93.66%
FV53	42047956	6.29	97.91%	94.27%	51.83%	94.20%	93.83%
FV71	43177168	6.46	97.91%	94.44%	51.48%	93.76%	93.42%
FV72	45950150	6.88	97.71%	93.79%	51.50%	93.52%	93.22%
FV73	43554558	6.51	98.06%	94.74%	51.44%	94.25%	93.88%
FV91	45147002	6.76	97.95%	94.40%	51.41%	94.87%	94.51%
FV92	42976550	6.42	97.95%	94.40%	51.33%	94.23%	93.95%
FV93	44756660	6.69	98.03%	94.62%	51.42%	94.39%	94.01%

**Table 2:** The expression levels of genes involved in fumonisin production.

Gene	Accession	Log 2 Fold Change			Annotation
		FV3 vs. FV5	FV5 vs. FV7	FV7 vs. FV9	
*FvZBD1*	FVEG 00314	−2.38 *	−0.84	1.18 *	PKS_ER domain-containing protein
*FvCpsA*	FVEG 00488	−0.46 *	0.26 *	−0.15	Glycosyltransferase 2-like domain-containing protein
*FvVelB*	FVEG 01498	−1.03 *	0.14	0.03	Velvet complex subunit 2
*Mads1*	FVEG 01965	−0.48 *	0.81 *	0.55 *	MADS-box transcription factor
*AREA*	FVEG 02033	−0.83 *	−0.04	−0.37 *	Nitrogen regulatory protein areA
*ART1*	FVEG 02083	−3.07 *	0.22 *	0.39 *	Beta-fructofuranosidase
*FvGbb2*	FVEG 02582	−0.74 *	−0.57 *	0.71 *	Guanine nucleotide-binding protein subunit beta-like protein
*Fvs.tuA*	FVEG 02853	−0.02	−0.36 *	0.88 *	Cell pattern formation-associated protein StuA
*FvAtfA*	FVEG 02866	0.93 *	0.42 *	−0.20 *	BZIP domain-containing protein
*Mads2*	FVEG 03759	−0.42 *	0.20	0.13	MADS-box transcription factor
*FUG1*	FVEG 04008	−1.71 *	0.61 *	0.58 *	DUF1752 domain-containing protein
*FvBCK1*	FVEG 05000	−0.26	0.35 *	0.11	STE/STE11/BCK1 protein kinase
*FvMk1*	FVEG 05063	−0.20	−0.07	1.39 *	Mitogen-activated protein kinase
*Lae1*	FVEG 05214	0.18	0.54 *	0.60 *	Sexual development regulator velC
*GBP1*	FVEG 05343	−0.01	0.08	0.368 *	Ribosome-interacting GTPase1
*PAC1*	FVEG 05393	−0.91	1.55 *	0.26 *	pH-response transcription factor pacC/RIM101
*FLBA2*	FVEG 06192	−0.64	0.17	0.68 *	DEP domain-containing protein
*Fvs.ET1*	FVEG 07811	−0.09	−0.07	0.48 *	Histone-lysine N-methyltransferase
*Fvs.O*	FVEG 08055	−0.68	0.58 *	−0.38 *	WW domain-containing protein
*FST1*	FVEG 08441	−1.11	−3.13 *	−2.20 *	MFS transporter
*FLBA1*	FVEG 08855	−1.19 *	−0.14	−0.66 *	Regulator of G protein signaling-like protein
*Sge1*	FVEG 09150	−1.50 *	−0.93 *	0.81 *	Global transcription regulator sge1
*CPP1*	FVEG 09543	0.26	−0.72 *	0.14 *	Serine/threonine-protein phosphatase
*GBB1*	FVEG 10291	0.08	−0.60 *	0.01 *	Guanine nucleotide-binding protein subunit beta
*FCK1*	FVEG 11159	−0.10	0.42 *	0.54 *	CMGC/CDK/CDK8 protein kinase
*FCC1*	FVEG 11306	1.24 *	0.12	0.39 *	RNA polymerase II holoenzyme cyclin-like subunit

Note: The expression levels of these genes are represented by different colors, with red indicating upregulation and blue indicating downregulation. The asterisk indicates FDR < 0.01.

## Data Availability

The raw sequence data reported in this paper have been deposited in the Genome Sequence Archive [52] in the National Genomics Data Center [53], China National Center for Bioinformation/Beijing Institute of Genomics, Chinese Academy of Sciences (GSA: CRA032526), which are publicly accessible at https://ngdc.cncb.ac.cn/gsa/search?searchTerm=CRA032526.

## Supplementary Materials

The following supporting information can be downloaded at: https://www.mdpi.com/article/10.3390/microorganisms14010102/s1, Supplementary Figure S1: The radial mycelial growth (A) and dry biomass (B) of *F. verticillioides* at different cultivation periods; Supplementary Figure S2: The top 15 most enriched pathways of DEGs analyzed by KEGG; Supplementary Table S1: Primers used in this study; Supplementary Table S2: Llist of all differentially expressed genes; Supplementary Table S3: Functional enrichment analysis of DEGs.
