# Supplementary material for: Transcriptomic Analysis of Fusarium verticillioides Across Different Cultivation Periods Reveals Dynamic Gene Expression Changes
Source: Microorganisms. 2026 Jan 2;14(1):102. doi: 10.3390/microorganisms14010102 (PMC12843636; doi:10.3390/microorganisms14010102)
Supplement: Supplementary file 1 [file microorganisms-14-00102-s001.zip › Supplementary Material.pdf]

Supplementary Material

1.1 Supplementary Figures

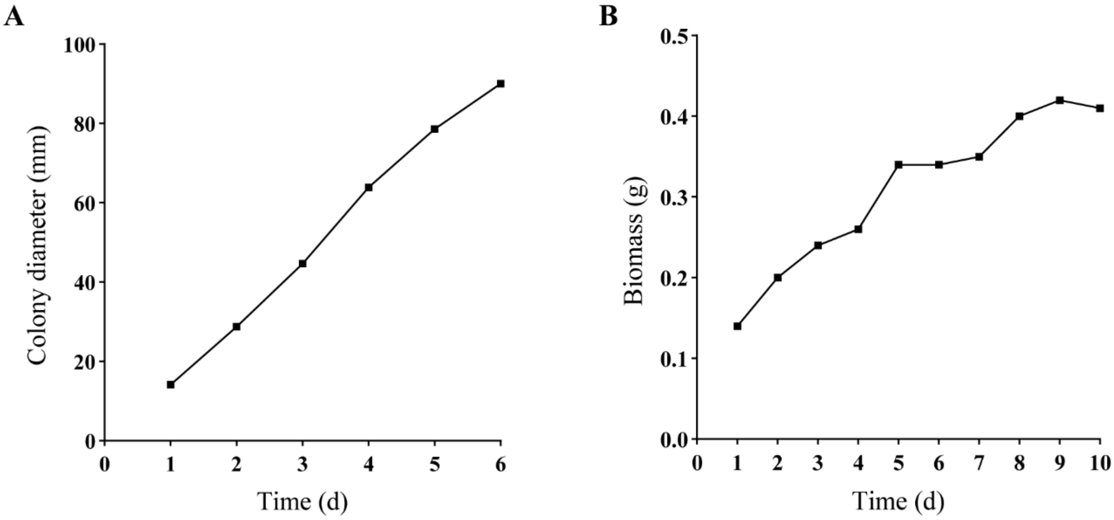

Supplementary Figure S1. The radial mycelial growth (A) and dry biomass (B) of *F.verticillioides* at different cultivation periods.

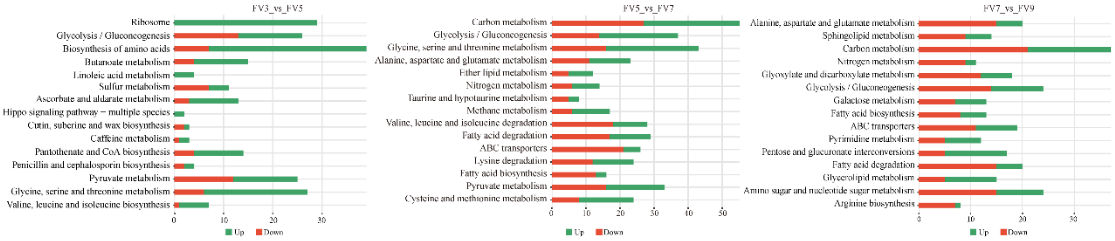

Supplementary Figure S2. The top 15 most enriched pathways of DEGs analyzed by KEGG.

Supplementary Table S1. Primers used in this study.

Gene	Primer name	sequences(5′ to 3′)
FVEG_00316	F35-316F	GGATTGAATTTCCGGGACGTG
	F35-316R	TCTAACCCGATGCCAACACC
FVEG_00319	F57-319F	GTCGAGGGCATCTATGGCAA
	F57-319R	AGAGCGACCAGTAAAGCACC
FVEG_00329	F35-329F	TCATGGGAACATGCTTGGAGT
	F35-329R	TCCAAGTACCGAAGAACACG
FVEG 00314	F79-314F	AGACCCAAGACATCGTGCAG
	F79-314R	GCTTTCGTTGAGGGTGGAGA
FVEG 08441	F57-441F	GTGGTATTGAGGACCGCGAA
	F57-441R	CCAAGGGAAGCGATGGTTCT
FVEG_12523	F35-523F	TCCGAGGTCATCTCACTGGT
	F35-523R	GATACTCGCTCACACGCTCA
FVEG_12521	F35-521F	GGCTGCTACAAGTCTACC
	F35-521R	TGATAATGCTGCGAAGTCT

FVEG_12533	F57-533F	GGCGTTTCGGCATTACGTC
	F57-533R	TGTTGGGCTTCCTGTGTGAG
FVEG_12534	F57-534F	GCGGAAACGACGGACTGTAA
	F57-534R	GTTGGAAGCTGTTGCTGTCG
FVEG_03344	F35-344F	CCGATCTTGGTCAGGGAACC
	F35-344R	AGCGGATCTGAATCTGGCTG
FVEG_10843	F35-843F	CGTCAATACCGAGGGCTACC
	F35-843R	CTGGTTCATGCTTCATGCGG
FVEG_16673	F57-673F	AGCCTTCAACAAGTACCAAGCC
	F57-673R	TCGAGCTTCCCATGACTTGT
FVEG_04436	F57-436F	ATGGCTACGCACAACCATGA
	F57-436R	GCTGCCTGTTTGGTTGAAGG
FVEG_05664	F79-664F	GCTACATCTGCTCCTGGCTT
	F79-664R	GCGCAGAGGTTGAGATCCAT
FVEG_08742	F79-742F	AAGGCTTACGTCACTGGTGG
	F79-742R	TGGCAGGCAAGTCAAACTCA
Actin	FV-Actin-F	TACACTTTCTCCACCACCGC
	FV-Actin-R	CATCGGGAAGCTCGTAGGAC
